# Increased diameter of arteries and veins in the caudal fins of *Danio rerio* *longfin ^t2^* fish is accompanied by fin overgrowth

**DOI:** 10.17912/micropub.biology.002008

**Published:** 2026-02-07

**Authors:** Jennifer S. Lanni, Benjamin W. Lyon, Stacy V. Nguyen

**Affiliations:** 1 Biological, Chemical and Environmental Sciences, Wheaton College - Massachusetts, Norton, MA, US

## Abstract

The classic dominant *longfin* zebrafish strain ( *lof ^t2^* ) has long fins due to ectopic expression of the potassium channel Kcnh2a in the fin mesenchyme. Potassium channel function is also known to affect the vasculature, but the fin vasculature has not been characterized in *lof ^t2^* fish. Here we show that *lof ^t2^* fish contain significantly increased artery and vein diameters in the caudal fin as compared to wildtype fish. Interestingly, these changes in blood vessel size are associated with areas of fin overgrowth, consistent with a possible role for the vasculature in fin size regulation.&nbsp;

**Figure 1. Artery and vein diameter in wildtype and lof t2 mutant zebrafish:**
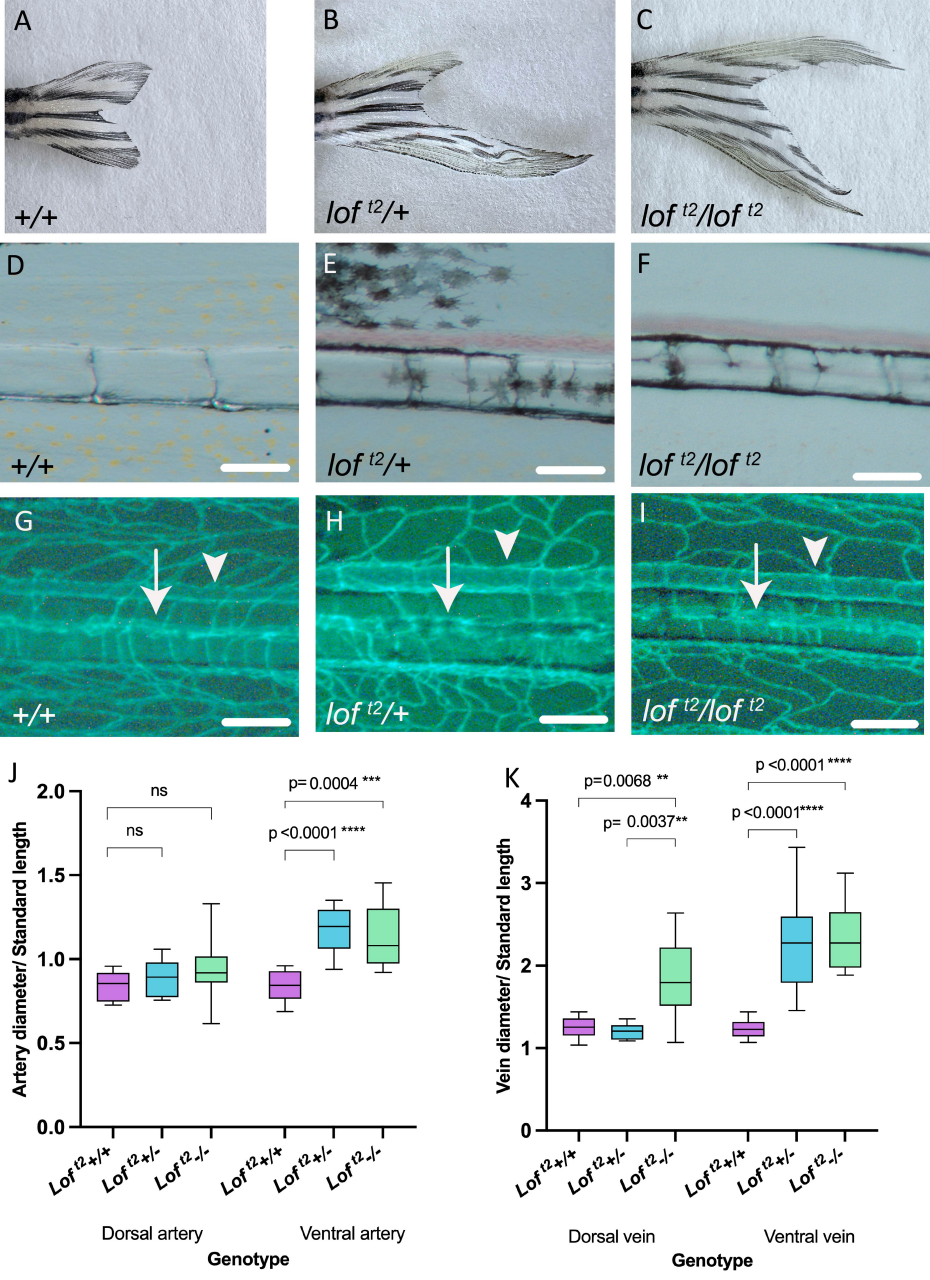
(A) *+/+* caudal fin. (B) *Lof ^t2^ /+* caudal fin with elongated ventral lobe. (C) *Lof ^t2^ /lof ^t2^* caudal fin with elongated dorsal and ventral lobes. (D-F) Brightfield microscopy of ventral lobe of caudal fin.&nbsp; (D) *+/+* ; (E) *Lof ^t2^ /+* ; and (F) *Lof ^t2^ /lof ^t2^* . Scale bar = 300 um. (G-I) Corresponding green fluorescent vasculature in *Tg(fli1a:EGFP)* ^y1^ ventral lobe of caudal fin. (G) *+/+* ; (H) *Lof ^t2^ /+* ; and (I)&nbsp; *Lof ^t2^ /lof ^t2^* . Arrow indicates intra-ray artery; arrowhead indicates primary vein flanking artery; scale bar = 300 um. (J) Genotype vs. artery diameter/standard length. (K) Genotype vs. vein diameter/standard length. For both J and K, an ANOVA was performed followed by multiple comparisons analysis using Tukey’s test. Line indicates mean; box indicates middle 50% of data points; error bars represent minimum to maximum values in data set. N.s not significant; ** p < 0.01; *** p < 0.001; **** p < 0.0001. *n* = 10 per genotype.&nbsp;

## Description

Zebrafish exhibit heritable variations in fin size that facilitate the study of growth regulation. These mutations primarily affect genes encoding proteins that mediate potassium transport, suggesting that ion regulation is critical in determining normal proportion (Silic & Zhang, 2023). In parallel, several mechanisms have been found to contribute to adult zebrafish caudal fin size and shape, including a calcineurin-mediated signaling pathway (Daane et al., 2018; Kujawski et al., 2014); the *Shh* pathway and *hox13* paralogs (Braunstein et al., 2021; Cumplido et al., 2024; Laforest et al., 1998; Surette et al., 2025); the *wnt10a* gene (Benard et al., 2024), and thyroid hormone (Harper et al., 2023). To date, only one of these mechanisms has been linked to potassium transport (Daane et al., 2018), leaving a gap in our understanding of how ion regulation can affect fin size.&nbsp;

Given the known effects of potassium on vascular tone (reviewed in (Dogan et al., 2019; Sahranavard et al., 2021), we wanted to assess whether the potassium channel mutations in long-finned zebrafish mutants affect the fin vasculature. Development of the vasculature has been closely studied in wildtype caudal and pectoral fins (Akiva et al., 2019; Bump et al., 2022; Leonard et al., 2023; Paulissen et al., 2022). Caudal fins are composed of 16-18 bony rays, each of which contains an artery and is flanked by two veins (Parichy et al., 2009; Tu & Johnson, 2011). The classic *longfin* zebrafish strain ( *lof ^t2^* ) has long fins due to ectopic expression of the potassium channel Kcnh2a in the fin mesenchyme (Daane et al., 2021; Stewart et al., 2021). We set out to characterize the caudal fin vasculature of adult *lof ^t2^* fish.

We crossed *lof ^t2^* mutant fish to transgenic fish expressing green fluorescent protein under the control of a pan-endothelial cell promoter ( *Tg(fli1a:EGFP)* ^y1^ (Lawson & Weinstein, 2002) and reared them to adulthood. As has been previously observed, fish homozygous for the *lof ^t2^* mutation have elongated fins, including the dorsal and ventral lobes of the caudal fin ( Figure 1C ). Fish heterozygous for the *lof ^t2^* allele display an elongated ventral caudal fin lobe but their dorsal caudal fin lobe maintains wildtype proportions ( Figure 1B ).&nbsp;

We imaged wildtype, *lof ^t2^* /+, and *lof ^t2^* / *lof ^t2^* &nbsp;adult caudal fins using brightfield and fluorescence microscopy. Enlarged blood vessels were evident in the ventral caudal fins of both homozygous and heterozygous fish, typically at the third fin ray from the exterior (Figures 1E, 1F). Figures 1G-I show representative images of arteries (arrows) and veins (arrowheads). In *lof ^t2^* /+ heterozygous fish, arteries and veins were significantly larger in diameter in the elongated ventral lobe, but no enlarged vessels were detected in the normally proportioned dorsal fin lobe (Figures 1J, 1K). In *lof ^t2^* / *lof ^t2^* homozygous fish, the arteries in the ventral fin lobe had increased diameter when compared to wildtype fish, as did the veins in both the dorsal and ventral fin lobes (Figures 1J, 1K).&nbsp;

We have found that *lof ^t2^* /+ and *lof ^t2^* / *lof ^t2^* fish display increased diameter of their caudal fin blood vessels and hypothesize that this phenotype is caused by potassium-mediated changes in vascular tone. Kcnh2a is a voltage-gated potassium channel that allows potassium to flow out of the cell (Sanguinetti et al., 1995; Warmke & Ganetzky, 1994). Overexpression of Kcnh2a should increase potassium transport out of fin mesenchymal cells and thereby elevate potassium concentration in the extracellular fluid surrounding the blood vessels. Even slight increases in potassium have been shown to cause hyperpolarization of vascular smooth muscle cells, muscle relaxation, and vasodilation (Haddy et al., 2006; Hein et al., 2025; Sobey, 2001). Blood vessel diameter is also maintained by endothelial cell shape and size (Diwan et al., 2025; Sugden et al., 2017) and may be affected by potassium concentration, as increased potassium causes swelling and softening of endothelial cells (Oberleithner et al., 2009). Future investigations should examine both endothelial cell morphology and blood vessel flow rates in longfinned and wildtype zebrafish.&nbsp;

The vasculature may also be an important factor in regulation of zebrafish fin growth. Blood vessel diameter and fin size are associated in *lof ^t2^* /+ heterozygous fish, in which only the overgrown ventral lobe has enlarged blood vessels. In wildtype zebrafish, endothelial cells are required during both larval development and fin regeneration for fins to reach their expected size (Bump et al., 2022; Bayliss et al., 2006). One limitation is that our experiments do not address potential causality between vessel size and fin overgrowth; this could be addressed in the future by treating fins with vasoactive drugs and assessing impact on fin size. It will also be of interest to determine whether changes in blood vessel diameter and flow are a common feature of other zebrafish fin size mutants. Interestingly, the longfinned zebrafish mutant *slc12a7a/kcc4a* has been shown to have larger vein diameter and decreased rate of blood flow in the caudal fin (Lanni et al., 2019). Taken as a whole, our findings suggest that potassium-induced changes in the vasculature of *lof ^t2^* zebrafish may contribute to fin overgrowth.&nbsp;

## Methods

*Zebrafish Husbandry and Maintenance*

The following zebrafish lines were used: *lof ^t2^* , *Tg(fli1a:EGFP)* ^y1^ , and wild-type AB. *lof ^t2^* was obtained by breeding the Tüpfel long fin strain ( *lof ^t2^ ; leo ^t1^* ) from Zebrafish International Resource Center onto an AB background and selecting for long fins. Crosses were performed such that each animal had only a single copy of the Tg(fli1a:EGFP)y1 transgene. Zebrafish were kept in a recirculating aquatic system and were maintained, bred and reared according to standard protocols (https://zfin.org). All procedures were conducted following established protocols and in full compliance with the Wheaton College Institutional Animal Care and Use Committee guidelines (Protocol #181-2025).&nbsp;

*Fin imaging&nbsp;*

Adult fish were anesthetized in 0.2% Tricaine in system water until immobile and standard length was recorded (mean standard length 29.5 mm; standard deviation 2.3 mm). Whole caudal fin photos were taken using an iPhone 14 Pro. Caudal fins were imaged using a Nikon SMZ800 dissecting microscope equipped for fluorescence detection and a Spot Insight 2 camera with Spot software.&nbsp;

*Blood vessel measurement*

Fin images were analyzed using Fiji (http://fiji.sc). Image brightness and contrast was adjusted if needed using Photoshop (Adobe). Blood vessel diameter was measured for the major artery running through the third-most exterior fin ray and from the paired vein directly below it (dorsal caudal fin lobe) or above it (ventral caudal fin lobe). Blood vessel diameter was measured in five positions in the region proximal to the first branch point of the third fin ray. If a visibly enlarged blood vessel was noted in ray 2 or 4, the vessels in that ray were chosen for measurement instead. Data were collected from ten fish of each genotype and used to calculate mean diameter for each blood vessel.

*Statistical analysis*

Statistical analysis and data visualization was performed using GraphPad Prism 10. One-way ANOVA was performed separately on artery and vein diameters followed by post-hoc Tukey’s HSD test. A p-value of ≤0.05 was considered significant (*) in all analyses.

## Reagents

**Table:** 

**STRAIN**	**GENOTYPE**	**AVAILABLE FROM**
AB	*Danio rerio*	Zebrafish International Resource Center
y1Tg	*Tg(fli1a:EGFP)* ^y1^ &nbsp;	Zebrafish International Resource Center
Tüpfel long fin (TL)	* lof ^dt2^ ; leo ^t1^*	Zebrafish International Resource Center
